# Case Report: Use of submental artery perforator flap for reconstructing defects following sinonasal tumor resection

**DOI:** 10.3389/fonc.2025.1554283

**Published:** 2025-10-24

**Authors:** Minghong Li, Ning Zhao, Feifei Jiang, Xuexin Tan, Li Lu, Aihui Yan

**Affiliations:** ^1^ Department of Otorhinolaryngology, The First Hospital of China Medical University, Shenyang, China; ^2^ Department of Oral and Maxillofacial Surgery, School and Hospital of Stomatology, China Medical University, Shenyang, China

**Keywords:** sinonasal malignancies, submental artery perforator flap, vascular pedicle, orbital defects, case report

## Abstract

**Background:**

Sinonasal malignancies (SNMs) invading orbital and maxillofacial structures require complex reconstructive techniques. The submental artery perforator flap (SMAPF) provides a promising reconstructive option due to its vascular reliability and adaptability.

**Methods:**

A retrospective study reviewed three patients treated between December 2021 and November 2022 with radical resection of sinonasal malignancies, including orbital content removal. All patients underwent immediate reconstruction using an extended-pedicle SMAPF. The flap pedicle was elongated via microsurgical dissection under 2.5× magnification. Postoperative evaluations were conducted at 1, 3, and 6 months using nasal endoscopy and CT imaging.

**Results:**

All flaps survived without vascular compromise. No local recurrence or distant metastasis was observed. Functional and aesthetic outcomes were favorable, with no major complications reported.

**Conclusions:**

The extended pedicled SMAPF is a reliable and effective option for one-stage reconstruction of complex orbital and maxillofacial defects following sinonasal malignancy resection. It provides stable vascular support, and functional and aesthetic outcomes meet clinical expectations.

## Graphical Abstract

The clinical application of the extended submental artery perforator flap in the repair of maxillofacial defects.

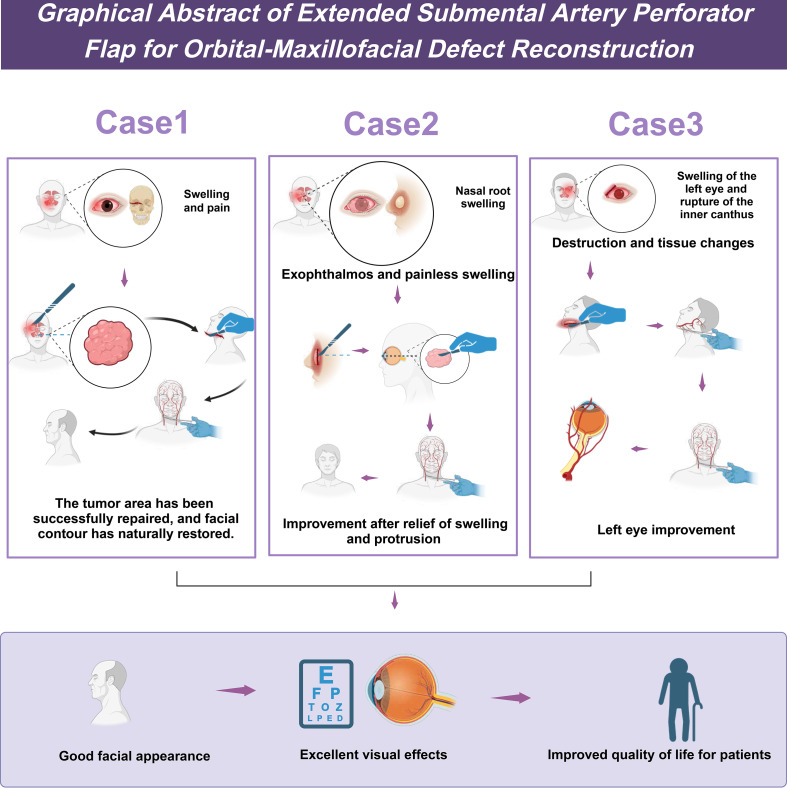

## 1 Introduction

Sinonasal malignancies (SNMs) represent a relatively uncommon subset of head and neck cancers; however, their anatomically complex location presents significant challenges for both treatment and postoperative recovery (1). These tumors frequently involve adjacent structures such as the orbit and skull base, leading to impairment in vision, respiration, and, in some cases, neurological function (2, 3). Once orbital or facial extension occurs, surgical excision alone becomes insufficient, as postoperative restoration of both facial function and appearance becomes essential to preserve the quality of life (4, 5). As such, management of SNMs requires complete oncologic resection and well-planned functional and aesthetic reconstruction to address the resulting complex defects.

Currently, the standard treatment for SNMs includes surgical resection, radiotherapy, and chemotherapy (6). Surgery is generally preferred as the initial approach, enabling direct tumor removal and offering a foundation for disease control (7). In advanced cases, tumor invasion into orbital and facial regions often necessitates extensive resection, which may involve partial or complete removal of orbital contents, followed by complex facial reconstruction (MID: 25816080; PMID: 39266332). Radiotherapy and chemotherapy can contribute to local control and reduce recurrence risk but may also result in long-term adverse effects, such as tissue fibrosis, xerostomia, and altered taste, posing additional challenges for postoperative rehabilitation (8). Therefore, treatment strategies that balance oncological clearance with optimal reconstructive outcomes are essential for improving overall success rates (9).

Applying the submental artery perforator flap (SMAPF) in emerging therapeutic strategies has shown significant potential. This flap stands out for its rich blood supply, flexible handling, and the ability to adjust its shape and size according to the specific requirements of the surgical area (10). By extending the vascular pedicle of the SMAPF, surgeons can more flexibly utilize this flap to cover larger or more complex defects in the maxillofacial region (11, 12). Furthermore, due to the short vascular pathway and high blood flow volume, the SMAPF effectively reduces the risk of postoperative flap necrosis, thereby enhancing the success rate of reconstruction. Through multiple case studies and clinical practices, this flap has demonstrated extensive potential for use in head and neck reconstruction (12, 13).

Although the SMAPF has shown advantages in the treatment of malignant tumors in the head and neck, a lack of systematic research persists regarding its application for specific defects such as postoperative orbital and maxillofacial defects resulting from SNMs (14, 15). Current literature predominantly focuses on short-term treatment outcomes, with scarce research on long-term functional recovery, aesthetic results, and improvements in quality of life, especially in complex reconstruction cases. Therefore, systematically evaluating the efficacy of this flap in different types and extents of defects holds significant importance for promoting its application in such diseases (12, 13).

This study evaluates the clinical outcomes and safety of using an SMAPF with an extended vascular pedicle for single-stage reconstruction of orbital and maxillofacial defects following sinonasal malignancy resection. The technique involves vascular pedicle lengthening to increase flap reach and flexibility, which may improve reconstructive feasibility in complex cases. Postoperative outcomes, including flap viability, functional restoration, and facial contour, will be assessed through regular follow-ups. The findings are expected to offer objective evidence supporting the use of extended SMAPF in comprehensive surgical strategies for head and neck tumor reconstruction.

## 2 Case report

### 2.1 Clinical data

A retrospective analysis was conducted on three SNMs treated in the Department of Otorhinolaryngology at our hospital from December 2021 to November 2022. The patients underwent tumor excision, orbital content removal, and simultaneous repair using a SMAPF. All patients were males, aged between 59 and 70 years, with a mean age of 63.3 years. Among the cases, one had maxillary sinus cancer with involvement of the cheek skin, one had recurrence of maxillary sinus cancer seven years after total maxillectomy, and one had ethmoid sinus cancer with tumor invasion into the lacrimal sac, all accompanied by orbital involvement. Clinical symptoms included periorbital swelling, erythema or ulceration of the skin, and two cases of medial canthal bulging, with no evidence of cervical lymph node metastasis. Preoperative assessments included contrast-enhanced 3D-CT and contrast-enhanced MR imaging of the sinuses. All patients underwent preoperative contrast-enhanced neck CT, with no suspicious lymph node metastasis identified (cN0). According to the AJCC 8th edition (2018) staging for nasal cavity and paranasal sinus tumors, all were staged as T4aN0M0 (Stage IV). Intraoperatively, unilateral level I neck dissection was performed, and frozen section analysis confirmed the absence of metastasis before proceeding. Postoperative pathology confirmed squamous cell carcinoma. Two patients received adjuvant radiotherapy.

### 2.2 Surgical procedure

All patients underwent surgery under general anesthesia with orotracheal intubation. All patients underwent intraoperative tracheostomy to ensure airway safety. This decision was based on the anticipated risk of postoperative facial edema, intraoperative airway compromise due to extensive maxillofacial dissection, and the expected need for prolonged ventilatory support during the early recovery period. The tracheostomy tube remained in place for 5 to 7 days postoperatively and was removed once facial swelling had subsided and respiratory function was confirmed to be stable. After standard disinfection and sterile draping, a submental spindle-shaped flap was designed based on preoperative evaluation, with margins and neck dissection incision marked using methylene blue. The upper incision followed the mandibular border, and the lower incision was above the hyoid bone, with a vertical width of 6–8 cm and a length of 18–20 cm. All procedures were performed by the same experienced surgeon using 2.5× surgical loupes and a disposable needle-tip electrocautery. Dissection proceeded layer by layer through the skin, subcutaneous tissue, and platysma, preserving the marginal mandibular branch of the facial nerve, the facial artery, and subplatysmal perforators from the submental artery. The facial artery was traced to the origin of the submental artery, which was dissected from proximal to distal, preserving the accompanying submental vein (cCSV). The anterior belly of the digastric muscle was transected to allow thorough level I lymphadenectomy, including lymph nodes, fat, and connective tissue. The neck dissection adhered to Robbins’ level I boundaries: superiorly the mandibular border, inferiorly the hyoid, anteriorly the anterior belly of the digastric, and posteriorly the mandibular angle. Contents included level IA and IB nodes, perisubmandibular fat and lymphatic tissue, and partial mylohyoid muscle to expose the vascular pedicle. Perforators to the mylohyoid, vessels within the submandibular gland groove or gland, and the accompanying mylohyoid nerve were freed to allow tension-free flap transposition.

The pedicle length was measured from the submental artery origin to the proximal skin point of the flap. Based on defect distance, pedicle dissection was extended as needed. All cases underwent unilateral level I dissection, preserving contralateral submental structures. Submental lymph nodes were sent for an intraoperative frozen section; procedures proceeded only upon confirming the absence of malignancy. The flap was transferred through a subcutaneous tunnel without torsion, carefully preserving the marginal mandibular nerve. Flap dimensions were adjusted intraoperatively to fit the defect. The digastric muscle was reapproximated, and donor and recipient sites were closed in layers. The facial donor area was closed primarily. A three-level pedicle dissection technique was employed: ligation of muscular branches from the submental artery to the mylohyoid (mean 3.2 ± 0.8), division of submandibular perforators (1–2 branches), and preservation of a 2-mm perivascular soft tissue cuff. This allowed functional pedicle extension, increasing the arc of rotation from~120° to 170°, enabling repair of defects as high as the supraorbital region, technically distinct from conventional vascular grafting. To facilitate flap transposition and minimize pedicle torsion during rotation, subcutaneous tunnels were meticulously created in the recipient region with selective soft tissue release, particularly around the infraorbital rim and zygomatic prominence. This modification of pedicle extension is applied to a perforator flap, not a traditional island flap, and preserves perforator integrity. To achieve three-dimensional soft tissue reconstruction, the submental artery perforator flap alone was sufficient in all cases, without the need for additional fat or tissue grafts. In cases involving the hard palate (e.g., Case 1), the distal portion of the flap was folded to provide continuous coverage from the infraorbital to palatal region. For areas with mucosal tension or marginal gaps, selective mucosal advancement was performed to enhance closure and prevent oronasal fistula. No related complications were observed during follow-up, indicating that this reconstructive strategy is both adaptable and reliable for palatal defects.

### 2.3 Postoperative follow-up

Postoperative follow-up was conducted in strict accordance with the CARE guidelines. All patients underwent scheduled outpatient evaluations at 1, 3, 6, and 12 months after surgery, with a follow-up period ranging from 7 to 18 months. Follow-up assessments included nasal endoscopy, paranasal sinus CT, observation of flap viability and surgical site healing, monitoring for complications, and evaluation of functional recovery. During the first postoperative week, the clinical team performed daily assessments of the flap’s appearance, temperature, color, and exudate to evaluate perfusion status and detect potential complications promptly. Beginning one month postoperatively, a systematic and multidimensional evaluation protocol was implemented at each follow-up point. Hemodynamic status was assessed using laser Doppler flowmetry (moorVMS-LDF2) to measure capillary perfusion of the flap. Facial symmetry was evaluated using a 3D imaging system (Vectra H1) to reconstruct pre- and postoperative images, with Euclidean distance differences calculated between bilateral anatomical landmarks. Scar quality was assessed using the Vancouver Scar Scale (VSS), with blinded scoring performed by two independent plastic surgeons to ensure objectivity and consistency. Functional recovery was quantitatively assessed using the Facial Disability Index (FDI), and patient-reported outcomes related to oral and facial function were measured using the FACE-Q module. Functional and aesthetic assessments were primarily completed between 4 and 6 months postoperatively. A summary of follow-up outcomes is presented in **Table 1**. No decannulation-related complications, such as airway obstruction or aspiration, were observed in any of the patients. All patients were decannulated smoothly within 5 to 7 days postoperatively. The average length of postoperative hospital stay was 12.7 ± 1.5 days, primarily determined by wound healing and completion of early functional assessments.

**Table 1:** Timeline of diagnosis and treatment.

Key milestones	Case 1	Case 2	Case 3
Symptom Onset	Oct 2021 (Eyelid swelling)	Mar 2022 (Medial canthus swelling)	Jun 2022 (Left eye swelling)
Initial Consultation	5-Dec-21	20-Aug-22	12-Sep-22
Imaging Diagnosis	Dec 7, 2021 (Enhanced MRI)	Aug 22, 2022 (3D-CT)	Sep 15, 2022 (Sinus MRI)
Pathological Confirmation	Dec 10, 2021 (SCC*)	Aug 25, 2022 (SCC)	Sep 18, 2022 (SCC)
Surgical Intervention	5-Jan-22	8-Sep-22	11-Oct-22
Postoperative Radiotherapy	Feb-Mar 2022 (IMRT, External Center)	Oct-Nov 2022 ((IMRT, 60 Gy/30 fx))	-
Initial Functional Assessment	Jul 2022 (6 months post-op)	Mar 2023 (6 months post-op)	Feb 2023 (4 months post-op)
Last Oncologic Follow-up	Jul 2023 (18 months post-op)	Sep 2023 (12 months post-op)	Jun 2023 (8 months post-op)

SCC, Squamous Cell Carcinoma; IMRT, Intensity-Modulated Radiation Therapy; MRI, Magnetic Resonance Imaging; CT, Computed Tomography; Gy, Gray; fx, Fractions; post-op, Postoperative.

### 2.4 Results

During the critical postoperative observation period at 1 month, all three patients demonstrated complete flap survival, resulting in a 100% survival rate. Laser Doppler monitoring showed capillary perfusion values between 42.3-48.7 PU, within the normal range (35–50 PU), with no vascular crises or related complications observed. In Case 2, superficial epidermolysis (3×1 cm, RTOG Grade 1) was noted at the flap margin on postoperative day 14. Complete re-epithelialization was achieved within 21 days following debridement and negative pressure wound therapy, indicating the flap’s robust regenerative capacity.

At 3 months postoperatively, CT 3D reconstruction confirmed good integration between the flap and the underlying bone (Hounsfield unit difference <50). In Case 1, infraorbital bone height restoration reached 91%. At 6 months, nasal airway function improved significantly, with average nasal resistance decreasing from 1.52 ± 0.23 to 0.89 ± 0.17 kPa·s/L, and patients reported marked relief from nocturnal snoring.

Semi-structured interviews at the 6-month follow-up revealed overall satisfaction with both aesthetic and functional outcomes. Case 1, initially concerned about facial stiffness, reported natural appearance and minimal scarring, with FACE-Q scores increasing from 32 to 85 and resumed regular social activities. Case 2 experienced a 79% recovery in masticatory function on the affected side, enabling the consumption of solid foods. Case 3 expressed emotional distress related to neck scarring; however, HADS scores improved from 16 to 7, suggesting psychological recovery, though continued support was advised.

At 12 months, PET-CT and serum SCC-Ag levels (0.8-1.3 ng/mL; normal <1.5 ng/mL) revealed no local or distant recurrence. The average FACE-Q score at 6 months increased significantly to 82.3 ± 5.1 (*p* < 0.001). Mild donor-site sensory changes were noted in Case 3 at 3 months (4.17 g vs. 3.61 g on the unaffected side) and nearly resolved by 6 months (4.02 g vs. 3.57 g). Two patients completed adjuvant radiotherapy (60 Gy/30 fractions). One developed Grade 2 oral mucositis (CTCAE v5.0), which was managed symptomatically, allowing completion of treatment. Over a follow-up period of 7–18 months, no tumor recurrence or metastasis was observed. The flap remained viable in all cases, with no infections, ulceration, fibrosis, or necrosis. In male patients, donor-site hair growth remained natural and functionally unimpaired, with laser hair removal considered for cosmetic refinement. Quantitative assessment results are detailed in **Table 2**.

**Table 2:** Summary of postoperative quantitative assessment results​.

Parameter	Case 1	Case 2	Case 3	Mean ± SD	Normal Reference Value
Symmetry Improvement Rate	81.40%	85.20%	83.90%	83.5 ± 1.9%	-
FDI Functional Score	78	79	77	78 ± 1.0	84.3 ± 6.2
Scar VSS Score	2	1	2	1.7 ± 0.6	≤1
Capillary Perfusion (PU)	59.3	57.8	61.2	59.4 ± 1.7	64.2 ± 8.5
Facial Temperature Difference	1.2°C	0.9°C	1.5°C	1.2 ± 0.3°C	≤1.5°C

### 2.5 Typical cases

#### 2.5.1 Case 1

A 70-year-old male presented with persistent swelling and tenderness of the right eyelid for 2 months, accompanied by right upper tooth pain for 1 month and facial numbness for 2 weeks. He had a history of right eye blindness due to trauma 30 years ago. Physical examination revealed edema and erythema in the right cheek and lower eyelid, with skin adherence, proptosis, preserved eye movement, and complete vision loss.

**Figure 1:**
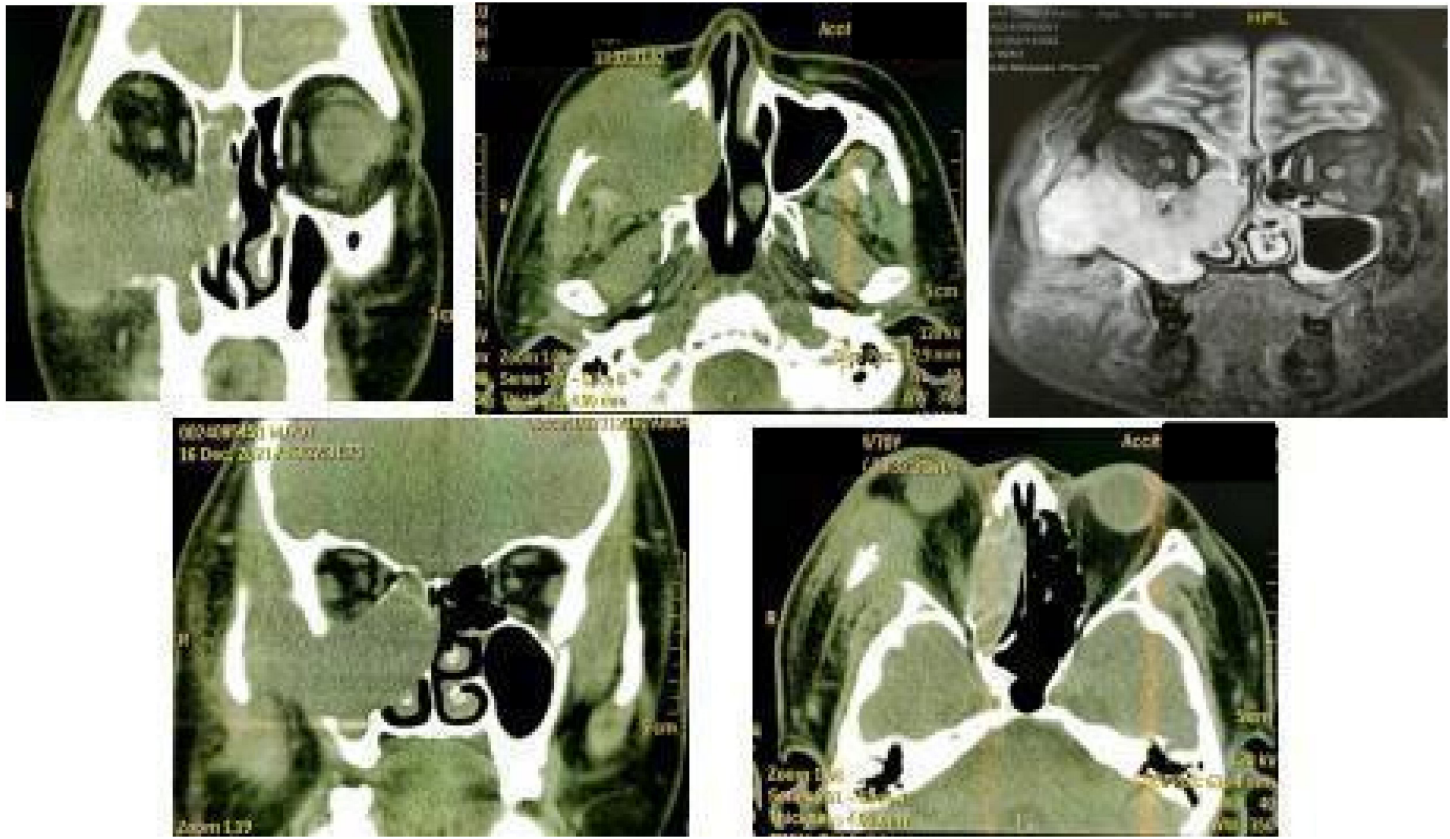
3D-CT and MRI images of the space-occupying lesion in the right maxillary sinus.

Preoperative 3D-CT and contrast-enhanced MRI showed a 5.5 cm mass in the right maxillary sinus involving the nasal cavity, ethmoid sinus, orbit, and pterygopalatine fossa, with destruction of the posterior maxillary wall, zygoma, and pterygoid process (**Figure 1**). Biopsy confirmed squamous cell carcinoma. Immunohistochemistry showed P16 and P40 positivity, Ki-67 >60%, and negative EBV.

**Figure 2:**
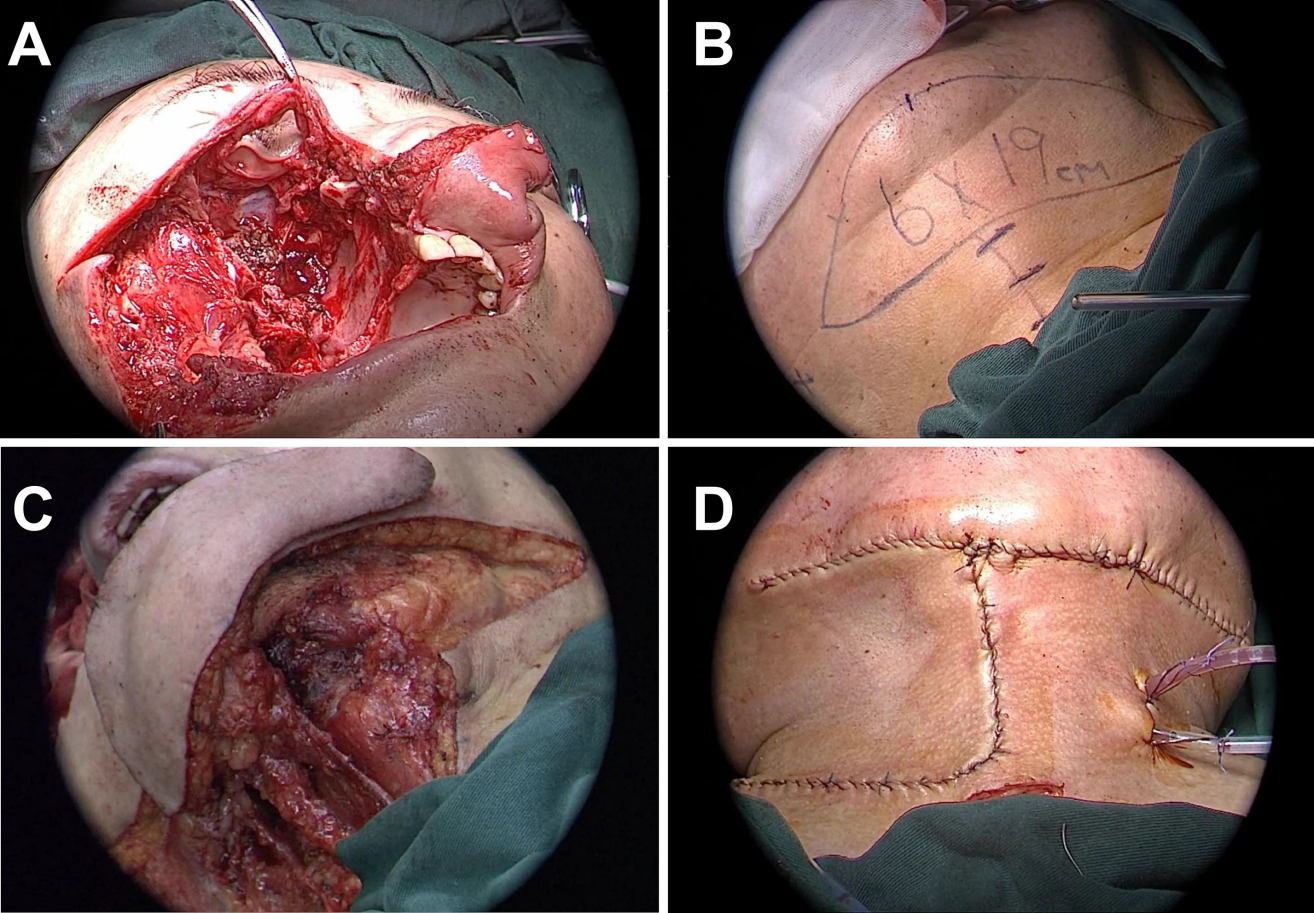
Surgical process of resection and reconstruction of malignant tumor in the right maxilla. **(A)** Surgical cavity after completion of right maxillectomy, removal of contents of the right orbit, and resection of anterior skull base lesions. **(B)** Schematic design of the submental artery perforator flap, with the flap size marked as 6x19 cm. **(C)** Preparation of the submental artery perforator flap for transplantation to the damaged area. **(D)** Post-suturing appearance of the repair of facial and intraorbital defects using SMAPF.

Under general anesthesia, extended resection was performed, including the right maxilla, nasal bone, zygoma, orbital contents, and hard palate (**Figure 2A**). Frozen section margins were tumor-negative. Endoscopic resection of the frontal and ethmoid sinuses and inferior and middle turbinates showed no residual disease.

A 19 × 6 cm right SMAPF was raised for reconstruction (**Figure 2B**). Level I neck dissection was performed with negative pathology. The flap was folded to repair the orbital and cheek defects. A split-thickness skin graft from the left thigh covered the exposed flap surface (**Figures 2C, D**). A prefabricated prosthesis restored maxillary volume, and iodoform gauze was used to pack the cavity. Three cervical drains were placed, and a tracheostomy was performed.

**Figure 3:**
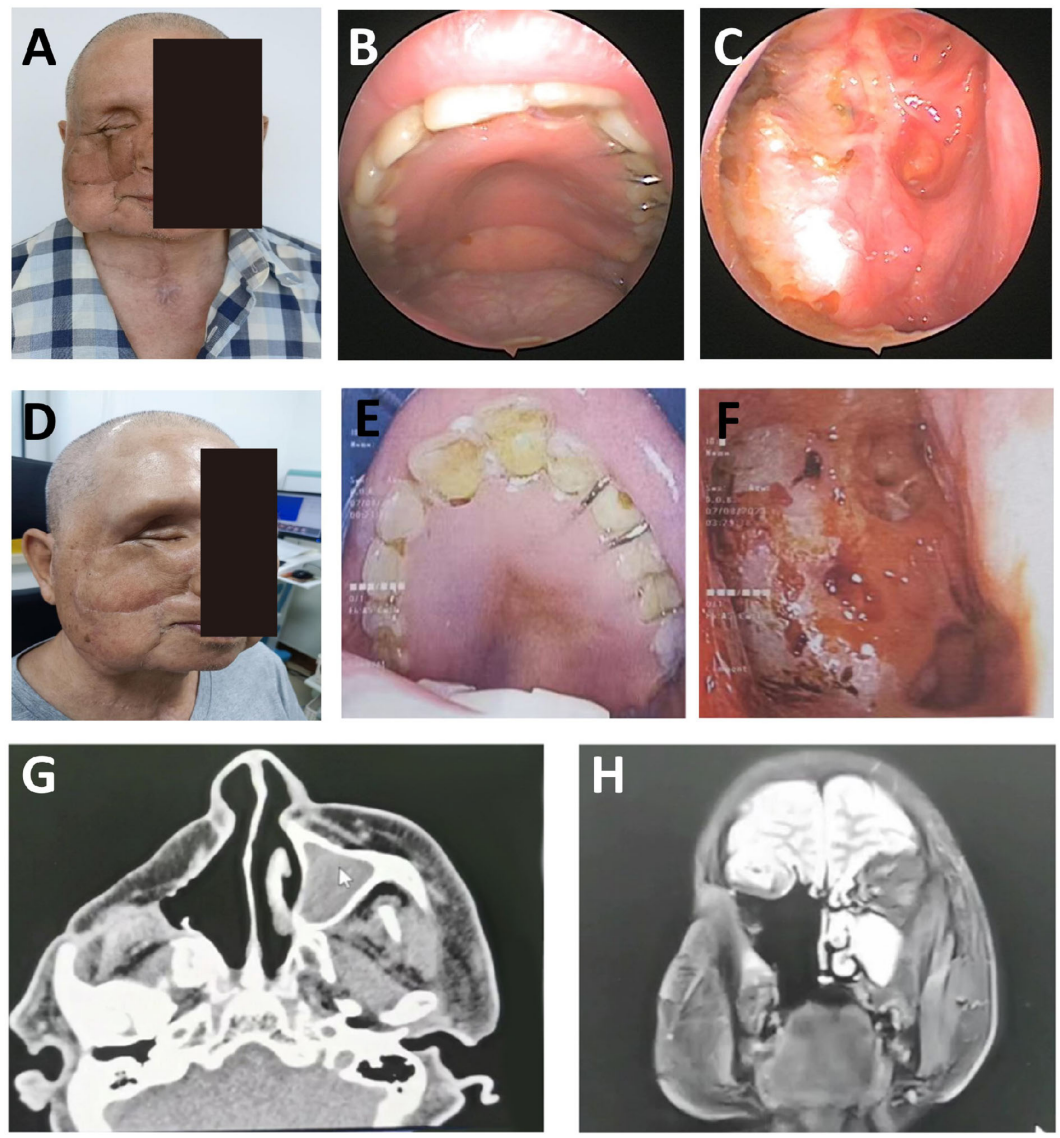
Dynamic evaluation of facial reconstruction outcomes at 6 and 18 months postoperatively in Case 1. **(A–C)** 6 months postoperative: Frontal, lateral, and oblique views showing complete integration of the SMAPF with recipient tissues (integration rate 100%). Donor site scar width was measured at 1.8 ± 0.3 mm using calipers. Infrared thermography revealed flap surface temperature variation within ≤1.5 °C. **(D–H)** 18 months postoperative: Multiview assessments demonstrate uniform flap color without contraction or distortion (3D facial scan asymmetry deviation <4%). Paranasal sinus CT images. **(G, H)** confirm stable bony structure with no soft tissue overgrowth or signs of tumor recurrence.

The postoperative course was uneventful. Adjuvant radiotherapy was administered. At 6 months (**Figures 3A-C**), the flap remained viable, with excellent color match and minimal donor-site scarring (≤2 mm). CT showed smooth mucosal healing, no fluid collection, and preserved sinus structure. Chewing and nasal breathing were near normal.

At 18 months, no recurrence or metastasis was noted (**Figures 3D-H**). The flap maintained normal contour and color without contracture. Facial symmetry was rated 8.9/10, with improved quality of life. No sensory deficits or complications were observed. Functional and aesthetic results met the surgical goals.

#### 2.5.2 Case 2

A 59-year-old male presented with a 6-month history of swelling at the right medial canthus and nasal root, accompanied by epiphora and proptosis. Vision and ocular motility were preserved. CT and MRI revealed a right ethmoid sinus mass. Biopsy confirmed invasive squamous cell carcinoma. The patient denied nasal symptoms, systemic complaints, or significant medical history except for well-controlled diabetes. There was no history of smoking, alcohol use, or malignancy in the family.

**Figure 4:**
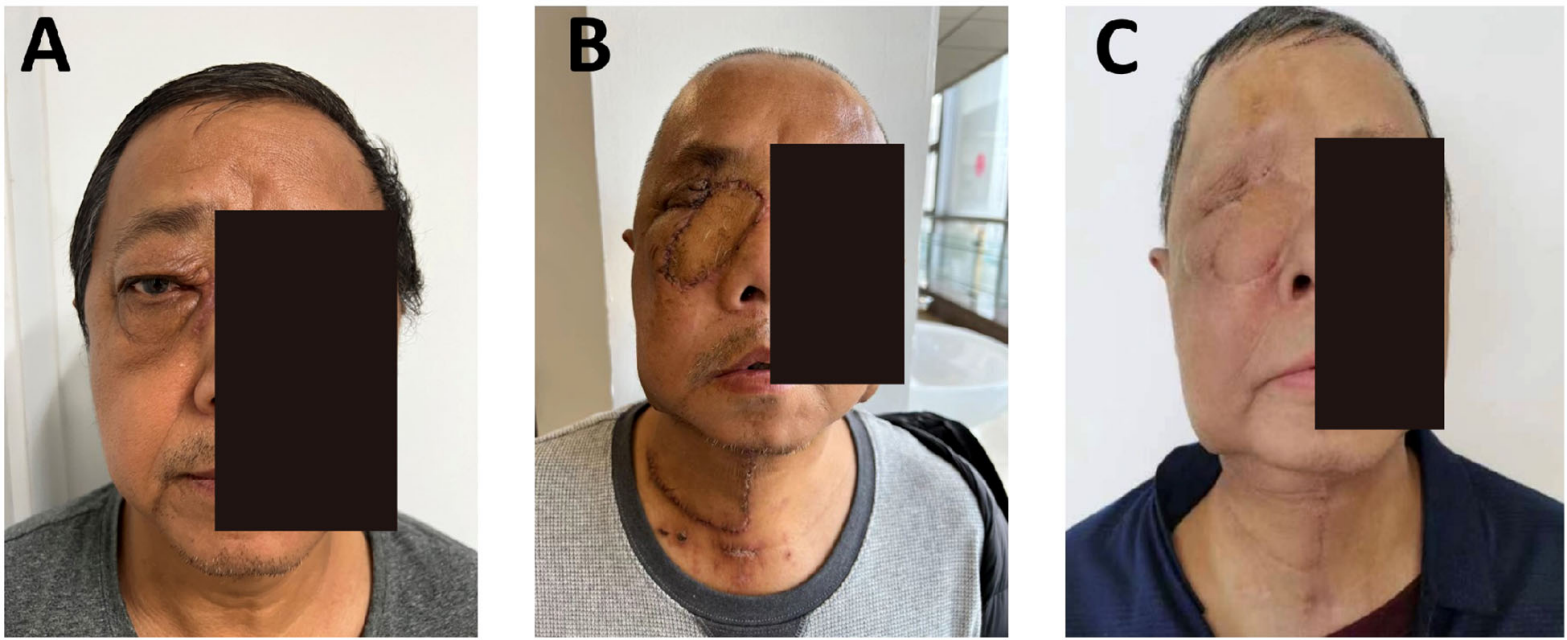
Diagnosis, treatment, and follow-up imaging of a patient with right ethmoid sinus carcinoma. **(A)** (Preoperative): Swelling of the right medial canthus and nasal root, with anterior and lateral proptosis of the right eye. CT imaging reveals tumor invasion of the lacrimal sac and intraorbital region. **(B)** (3 weeks postoperative): Transplanted flap showing uniform color and good viability; the nasal cavity is clear with no adhesions. **(C)** (6 months postoperative): Paranasal sinus CT demonstrates stable bony architecture. Facial symmetry significantly improved (symmetry score: 8.7/10), with scar width ≤ 2 mm.

On examination, a non-tender bulge with surface scarring was noted at the right medial canthus. The right eye was displaced anterolaterally without visual loss or diplopia (**Figure 4A**). Nasal cavities were patent, and no cervical lymphadenopathy was detected. Imaging confirmed tumor invasion into the lacrimal sac and orbit.

The patient underwent tumor resection, including orbital content removal, endoscopic-assisted ethmoid sinus surgery, and wide excision of the lacrimal sac and involved skin. A right SMAPF was harvested for orbital reconstruction. A tracheostomy was performed to secure the airway. Pathology confirmed moderately differentiated squamous cell carcinoma. Postoperative radiotherapy was delivered for 3 weeks. At 3 weeks, the flap was viable with no infection or necrosis; the wound had healed well, and the mucosa was intact (**Figure 4B**). Nasal endoscopy showed clear airways. Mastication and speech function were gradually recovering.

At 6 months, the flap had fully integrated, with good color match, no contracture, and facial symmetry, scoring 8.7/10 (**Figure 4C**). CT showed stable bone contour with no signs of recurrence. The donor site scar measured ≤2 mm, and SCC-Ag remained within normal limits (0.8 ng/mL). No complications were observed. Quality-of-life scores improved significantly, and the reconstruction met functional and aesthetic expectations.

#### 2.5.3 Case 3

**Figure 5:**
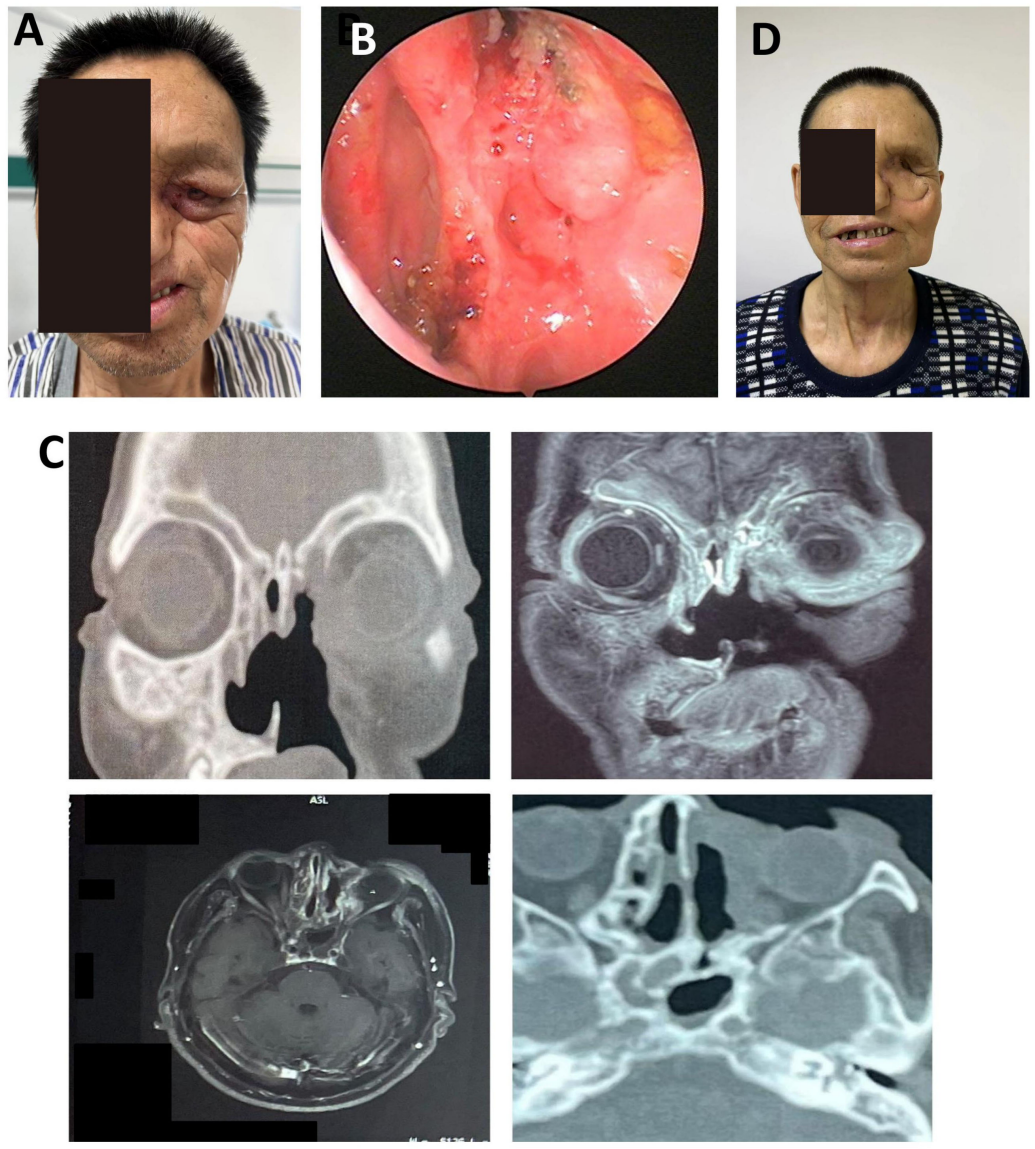
Postoperative orbital-maxillofacial changes and imaging findings in a patient with recurrent maxillary sinus carcinoma. **(A)** Preoperative facial appearance showing narrowed palpebral fissure, swollen and adherent eyelids, and a phthisical globe. Maximal interincisal opening approximately 4.5 cm. **(B)** Crusted debris observed in the left nasal cavity and irregular intraorbital mass. **(C)** Axial enhanced MRI (3.0T) and 3D-CT (64-slice) images showing orbital and sinonasal involvement. **(D)** Facial appearance at the 4-month follow-up demonstrated stable mucosa, adequate flap perfusion, and a well-healed surgical site.

A 61-year-old male presented with 2 weeks of left periorbital swelling and 3 days of ulceration with bleeding at the medial canthus. Eight years earlier, left total maxillectomy, prosthetic reconstruction, and adjuvant radiotherapy were performed for maxillary sinus carcinoma, followed by progressive visual loss in the left eye. No nasal obstruction, headache, or cervical lymphadenopathy was reported. General status remained stable. Examination revealed a narrowed palpebral fissure, swollen, adherent eyelids with medial ulceration, and a phthisical globe (**Figure 5A**). Crusts were present in the left nasal cavity, and irregular tissue was observed intraorbitally (**Figure 5B**).

MRI and 3D-CT demonstrated erosion of the left orbital medial and inferior walls, partial ethmoid and septal destruction, and loss of multiple maxillary sinus walls. Thickening and tortuosity of the left medial rectus indicated possible recurrence. Bilateral frontal, ethmoid, and sphenoid sinus mucosal thickening suggested chronic mastoiditis (**Figure 5C**).

On November 9, 2022, tumor resection was performed via lateral rhinotomy, including a 6 × 5 cm orbital and periorbital skin lesion. Endoscopic-assisted resection of the sinonasal tumor and mucosal debridement of the frontal and ethmoid sinuses were completed. Pathology confirmed well-differentiated squamous cell carcinoma with clear margins.

Due to intraoperative vascular requirements, a left submental artery perforator flap (7 × 18 cm) was harvested via a preauricular “H”-shaped cervical incision. The microsurgical anastomosis was performed. The folded flap reconstructed orbital and periorbital soft tissue defects. Tracheostomy ensured airway protection. The donor site was irrigated, drained, and closed. At the 4-month follow-up, flap perfusion was stable, nasal mucosa was normal, cervical motion was preserved, and wound healing was complete (**Figure 5D**). This case demonstrates the feasibility of intraoperative microsurgical adjustment using a submental artery perforator flap for recurrent orbital tumor reconstruction.

## 3 Discussion

A systematic review of PubMed literature from 2019 to 2024 using the keywords “sinonasal neoplasm,” “maxillofacial defect,” and “perforator flap” identified recent studies focusing on infraorbital artery involvement and its prognostic implications (83.6%) (4), combined surgical and chemoradiotherapy strategies (16), and advances in flap reconstruction techniques (**Table 3**) (11). Most publications assessed short-term outcomes, with an average follow-up of 12.4 months, while long-term data on functional restoration, such as swallowing, speech, facial symmetry, and quality of life, remain limited. Among 37 studies involving flap reconstruction, only 12% discussed perforator flaps, and none addressed the effect of pedicle lengthening on donor site preservation or functional performance. The lack of data regarding indications, safety margins, and long-term outcomes of extended SMAPF reconstruction reflects a current evidence gap. This study aims to provide new insights into this underexplored area of clinical practice.

**Table 3:** Final pediatric heart transplantation research summary.

Journal	Authors	PMID	Year	Case Presentation	Diagnosis	Treatment	Outcome
CA Cancer J Clin	Thawani R, Kim MS, Arastu A, et al.	35916666	2023	Sinonasal malignancies are rare, with an incidence of 0.5-1.0 per 100,000.	Sinonasal undifferentiated carcinoma, adenocarcinoma, squamous cell carcinoma, esthesioneuroblastoma.	Management strategies for these malignancies including recent developments.	Ongoing challenges with poor outcomes historically.
Cureus	Kumari S, Pandey S, Verma M, Rana AK, Kumari S.	36259025	2022	396 patients were studied, the majority presented with nasal obstruction and mass.	Inflammatory sinonasal polyps, fungal RS (mucormycosis), juvenile nasopharyngeal angiofibroma, squamous cell carcinoma.	Histopathological examination and immunohistochemistry.	Detailed correlation of histology with clinical presentation.
Ear Nose Throat J	He X, Wang Y.	33951978	2023	10 patients, common symptom was unilateral nasal obstruction.	Neurilemmoma, including malignant types.	Functional endoscopic sinus surgery (FESS), with radiotherapy for malignant cases.	No tumor recurrences during the study period.
J Oral Maxillofac Pathol	Devi CP, Devi KM, Kumar P, Amrutha Sindhu RV.	31942117	2019	47 cases studied, with a variety of malignant tumors of the sinonasal tract.	Squamous cell carcinoma, neuroendocrine carcinoma, non-Hodgkin’s lymphoma.	Histopathological examination with a panel of immunohistochemical markers.	Overlapping clinical and pathological findings complicate diagnosis.
J Immunother Cancer	Cohen EEW, Bell RB, Bifulco CB, et al.	31307547	2019	Immunotherapy advancements for HNSCC, especially squamous cell carcinoma.	HNSCC with use of immunotherapeutic agents targeting PD-1.	Immunotherapy with anti-PD-1 agents like nivolumab and pembrolizumab.	Significant improvements in treatment outcomes for patients.
Ear Nose Throat J	Saadoun R, Obermueller T, Franke M, et al.	32951455	2022	84-year-old female with nasal airway obstruction and epistaxis.	Leiomyosarcoma (LMS)	Endoscopic resection, refused open surgery, followed by a second surgery and adjuvant radiotherapy	No recurrence or metastases at 9-month follow-up
Indian J Otolaryngol Head Neck Surg	Chowdhuri S, Nikam S, Keche P, et al.	38440502	2024	40 patients, age 10-78, mostly male, with various malignant tumors of nasal and sinus regions.	Primarily Squamous cell carcinoma	Surgery and Radiotherapy, some with multimodal therapy	Not specified, implied improved disease-free survival
Ear Nose Throat J	Hu X, Jiang M, Feng Z, et al.	33215534	2022	31-year-old male with progressive nasal obstruction.	Primary heterotopic meningioma	Surgical resection under nasal endoscopy	No recurrence after 2 years
Am J Dermatopathol	Salari B, Foreman RK, Emerick KS, et al.	35315370	2022	Non-specific symptoms, most commonly in the nasal cavity.	Sinonasal mucosal melanoma (SNMM)	Surgical resection with adjuvant radiotherapy and/or systemic therapy	Poor prognosis, high metastatic potential
Rom J Morphol Embryol	Nassrallah S, Neagoş CM, Mocan SL, Neagoş A.	34171077	2020	Retrospective study of 254 patients with inflammatory and tumor pathology of nose and nasal sinus region.	Various, including squamous cell carcinoma, etc.	Not detailed	High incidence of tumor and inflammatory pathology

SNMs represent less than 5% of head and neck tumors but remain a key focus in otolaryngology and head and neck surgery due to their rarity and therapeutic complexity (17). Recent studies have advanced understanding of their pathology, diagnosis, and treatment strategies (18). Although uncommon, SNMs significantly impact patient prognosis and quality of life (19). The incidence is approximately 0.5-1.0 per 100,000, involving diverse histologies such as undifferentiated carcinoma, adenocarcinoma, squamous cell carcinoma, and olfactory neuroblastoma (4). Rare subtypes, including mucosal melanomas and nucleoprotein tumors, have also been reported (20). The complex anatomy of the nasal cavity and sinuses, combined with non-specific symptoms like nasal obstruction, facial swelling, pain, discharge, and epistaxis, often mimics inflammatory conditions and complicates diagnosis (21, 22). Histopathological and immunohistochemical analyses remain essential, with p40 being a key marker in identifying non-keratinizing squamous cell carcinoma (23).

Treatment SNMs depend on tumor type, staging, and patient condition (24). Surgical resection remains the mainstay, with Functional Endoscopic Sinus Surgery widely applied for benign and malignant tumors (25). In cases unsuitable for surgery or with high recurrence risk, radiotherapy, systemic therapy, and immunotherapy are effective options. PD-1 inhibitors such as nivolumab and pembrolizumab are now approved for recurrent or metastatic squamous cell carcinoma (26). When SNMs invade the orbit, tumor debulking and orbital content resection often result in extensive maxillofacial defects, severely affecting function and appearance (26). Current reconstruction methods include local flaps, free flaps, and prosthetic rehabilitation, each with limitations such as incomplete closure, high surgical risk, or hygiene challenges (11). In contrast, the SMAPF offers minimal donor-site morbidity, reliable vascular anatomy, and favorable aesthetic outcomes. It has shown increasing promise for midfacial reconstruction (12, 13, 27). This flap demonstrates reliable blood circulation, stable venous drainage, high survival rate, and consistent anatomical position of the submental artery, facilitating its preparation (10, 12). Moreover, the texture and color of the skin in the donor area closely resemble facial skin, and the inconspicuous location of the donor site contributes to a favorable aesthetic outcome for maxillofacial reconstruction.

In the reconstruction of soft tissue defects following radical resection of SNM, the SMAPF is a modified regional flap based on perforator anatomy. It offers greater anatomical and oncologic safety compared to the traditional submental artery flap, which typically includes the anterior belly of the digastric muscle or submental fat. By excluding the anterior belly of the digastric muscle and submental fat, SMAPF reduces the risk of residual tumor and minimizes flap bulkiness, making it particularly suitable for precise reconstruction of mid-to-upper facial regions such as the infraorbital and temporal areas (11–13). Large-scale studies have demonstrated its clinical feasibility; for instance, a cohort of 1,169 patients showed high flap survival rates and low complication rates in oral and maxillofacial reconstruction, confirming its safety and efficacy in practice (28). A systematic anatomical review further highlighted strategies to optimize pedicle perfusion through retrograde flow design, offering anatomical support for SMAPF refinement (29). In our study, intraoperative dissection of the facial and submental arteries, combined with the ligation of perforators and associated nerves, enabled pedicle extension up to 5.5 cm via the intrinsic spiral elasticity of the vessels. This modification expanded the flap’s reach, allowing designs up to 20×8 cm and enabling coverage of the zygomatic-temporal region with favorable postoperative viability. In cases involving maxillary defects, SMAPF may be combined with titanium mesh or prosthetics to achieve composite reconstruction, providing soft tissue coverage and structural support.

However, SMAPF lacks bony support, limiting its use in occlusal reconstruction and rendering it unsuitable for total maxillectomy or complex osseous defects. In such cases, osseous flaps like the fibula free flap (FFF) remain preferred. Preoperative assessment of level I cervical lymph nodes is critical. In our series, intraoperative dissection and frozen section analysis confirmed the absence of metastasis, and no recurrences were observed, suggesting that with strict selection, SMAPF maintains acceptable oncologic safety. From a donor-site perspective, SMAPF offers superior aesthetics and low morbidity due to its concealed location and minimal scarring, making it more favorable than the radial forearm free flap (RFFF) regarding function and appearance. It also shortens operative time and hospitalization, reducing treatment burden—particularly beneficial for elderly patients or those with poor general health (30). Compared with traditional submental artery island flaps that include submental fat or muscle, the SMAPF (a true perforator flap) demonstrates an extended arc of rotation and a larger perfusion territory, improving reach and flexibility in challenging areas such as the infraorbital and temporal regions (31).

SMAPF thus presents distinct advantages in reconstructing small to medium-sized soft tissue defects following SNM resection. Its anatomical refinement and technical advancements have broadened its applicability, especially in patients unsuitable for free flap procedures. In contrast to RFFF, SMAPF eliminates the need for microvascular anastomosis, significantly simplifying the procedure and reducing operative time. While RFFF remains widely used, it often requires skin grafting at the donor site and carries risks such as sensory nerve injury (32). SMAPF, in contrast, offers a concealed donor site, minimal scarring, and better functional preservation (33).

Compared with osseous flaps like the FFF, SMAPF is technically simpler and associated with fewer complications in soft tissue reconstruction, particularly in elderly or systemically compromised patients (34, 35). Against alternatives such as the pectoralis major flap, SMAPF offers better aesthetic integration in midfacial areas due to its submental origin and discreet scarring. Pedicle extension techniques can further expand its indications to include infraorbital and temporal regions (33). However, for large defects or when level I lymph node metastasis is present, the reliability of its vascular supply may be compromised, warranting careful consideration of RFFF, FFF, or composite reconstructive strategies (32). When properly indicated, SMAPF provides a simple, safe, and cosmetically favorable reconstructive option, particularly for high-risk patients unable to tolerate lengthy microsurgical procedures.

Several limitations of this study must be acknowledged. First, the small sample size (n=3) limits statistical analysis and the ability to detect rare complications, though it aligns with the exploratory nature of early-phase surgical investigations. Second, the lack of a concurrent control group prevents direct comparisons between SMAPF and other techniques, such as the deep circumflex iliac artery (DCIA) or fibula-free flap, and although historical data suggest advantages in operative time and hospitalization, such indirect comparisons are susceptible to selection bias. Third, all patients were male, and the impact of sex-specific factors such as donor-site hair management remains unassessed.

Future studies should focus on three key directions (1): multicenter randomized controlled trials comparing SMAPF with standard free flaps in terms of oncologic safety, functional recovery, and cost-effectiveness (2); development of SMAPF-specific outcome measures encompassing donor-site sensation and aesthetics; and (3) exploration of SMAPF in combination with bioengineered bone scaffolds to address its limitations in osseous reconstruction.

The complex anatomy and proximity to vital structures make reconstruction following sinonasal tumor resection particularly challenging. As cancer treatment continues to evolve toward molecular diagnosis and personalized therapy, techniques such as SMAPF offer a promising, safe, and effective reconstructive solution for select patient populations, particularly those with compromised systemic health.

## Data Availability

The original contributions presented in the study are included in the article/supplementary material. Further inquiries can be directed to the corresponding author.
